# Effectiveness of Serious Games to Increase Physical Activity in Children With a Chronic Disease: Systematic Review With Meta-Analysis

**DOI:** 10.2196/14549

**Published:** 2020-04-01

**Authors:** Daniël Bossen, Aline Broekema, Bart Visser, Annette Brons, Annieck Timmerman, Faridi van Etten-Jamaludin, Katja Braam, Raoul Engelbert

**Affiliations:** 1 ACHIEVE Center of Applied Research Faculty of Health Amsterdam University of Applied Sciences Amsterdam Netherlands; 2 Digital Life Centre Faculty of Digital Media and Creative Industries Amsterdam University of Applied Sciences Amsterdam Netherlands; 3 Topsport Amsterdam Amsterdam Netherlands; 4 Amsterdam University Medical Center Medical Library University of Amsterdam Amsterdam Netherlands; 5 Amsterdam University Medical Center Rehabilitation University of Amsterdam Amsterdam Netherlands; 6 Amsterdam University Medical Center Pediatrics University of Amsterdam Amsterdam Netherlands

**Keywords:** video games, computer games, pediatrics, chronic disease, exercise therapy, health education

## Abstract

**Background:**

Physical activity (PA) is important for children with a chronic disease. Serious games may be useful to promote PA levels among these children.

**Objective:**

The primary purpose of this systematic review was to evaluate the effectiveness of serious games on PA levels in children with a chronic disease.

**Methods:**

PubMed, EMBASE, PsycINFO, ERIC, Cochrane Library, and CINAHL were systematically searched for articles published from January 1990 to May 2018. Both randomized controlled trials and controlled clinical trials were included to examine the effects of serious games on PA levels in children with a chronic disease. Two investigators independently assessed the intervention, methods, and methodological quality in all articles using the Cochrane risk of bias tool. Both qualitative and quantitative analyses were performed.

**Results:**

This systematic review included 9 randomized controlled trials (886 participants). In 2 of the studies, significant between-group differences in PA levels in favor of the intervention group were reported. The meta-analysis on PA levels showed a nonsignificant effect on moderate to vigorous PA (measured in minutes per day) between the intervention and control groups (standardized mean difference 0.30, 95% CI –0.15 to 0.75, *P*=.19). The analysis of body composition resulted in significantly greater reductions in BMI in the intervention group (standardized mean difference –0.24, 95% CI –0.45 to 0.04, *P*=.02).

**Conclusions:**

This review does not support the hypothesis that serious games improve PA levels in children with a chronic disease. The meta-analysis on body composition showed positive intervention effects with significantly greater reductions in BMI in favor of the intervention group. A high percentage of nonuse was identified in the study of serious games, and little attention was paid to behavior change theories and specific theoretical approaches to enhance PA in serious games. Small sample sizes, large variability between intervention designs, and limited details about the interventions were the main limitations. Future research should determine which strategies enhance the effectiveness of serious games, possibly by incorporating behavior change techniques.

## Introduction

Worldwide, there are many children who have been diagnosed with a chronic disease [1,2]. A disease or condition is considered chronic in childhood if all 4 of the following conditions are met: (1) it occurs in children aged 0-18 years; (2) the diagnosis is based on medical scientific knowledge and can be established using reproducible and valid methods or instruments according to professional standards; (3) it is not (yet) curable or, for mental health conditions, it is highly resistant to treatment; and (4) it has been present for >3 months; it will, very probably, last >3 months; or it has occurred ≥3 times during the past year and will probably recur again [3]. Having a chronic disease during childhood can impact all elements of growth and development, including physical, psychosocial, and emotional functioning [4]. Physical activity (PA) is important for general health and to minimize the impact of chronic diseases on children. Unlike healthy children, children with chronic diseases are more often restricted in PA, such as playing, running and leisure activities. As a consequence, children with a chronic disease are less physically active than their healthy peers [5,6]. Participation in PA is of particular importance for children with a chronic disease [7-12]. In children with a chronic disease with a physical cause such as juvenile idiopathic arthritis, PA can improve muscle strength and physical fitness without exacerbating joint pain [8]. And, for children with type 1 diabetes mellitus, a physically active lifestyle is beneficial for glycemic control [7] and insulin sensitivity [11]. In addition, participation in sports and PA improves social functioning and mental health for all children [10].

To increase PA levels in children with a chronic disease, various physical exercise interventions have been developed, but not all have had substantial and significant effects [13-15]. The growing popularity of videogames has led to the development of serious games to promote PA in children. Serious games are especially suitable for children as these interventions use interactive and visual strategies that match the learning style of these ‘digital natives’ [16]. Well-designed games are adjustable in content, provide a feeling of satisfaction, and are challenging; they match the personal interest, motor skills, and cognitive levels of children [16]. Serious games are accessed through a personal computer, game console, tablet, or a smartphone, all of which are commonly used in a general family’s daily life. According to Bergeron [17], serious games are defined as interactive computer applications, with or without a hardware component, that have challenging goals, are fun to play, are engaging, incorporate concepts of scoring, and impart skills, knowledge, or attitudes to the user that can be applied in the real world. Whereas the hardware is comparable between games, the program itself and working mechanisms may differ. For example, Dance Dance Revolution (Konami of America, Inc., Redwood City, CA) is an exergame available on different platforms that uses cameras, motion sensors, and force sensors to encourage dancing. Other games, such as Reumaatjes@work, motivate and educate children through an interactive website with films, puzzles, and brain twisters to better cope with having childhood juvenile idiopathic arthritis and promote PA [18].

According to previous reviews, serious games have the potential to promote a physically active lifestyle in children. Especially in studies assessing the effectiveness of serious games in overweight children, first results indicate that serious games can help increase energy expenditure, heart rate, and program compliance [19]. Other studies have shown improvements in specific health outcomes, such as lung function and glycated hemoglobin A1C [20,21]. The evidence from reviews in healthy children and adolescents also supports the argument that serious games effectively promote quantitative [22-25] and qualitative PA. [26,27]. However, the long-term effectiveness on PA maintenance is not well known [22-24].

Despite the potential of serious games for healthy and overweight children, less is known about the effectiveness of serious games that promote PA in children with a chronic disease. Compared with healthy and overweight children, children with a chronic disease have unique perceived barriers and other perspectives and desires with respect to PA and serious games. In children with juvenile idiopathic arthritis, for example, pain has a major impact on the performance of PA in daily life [28], while children with diabetes have a fear of hypoglycemia [29]. It would be worthwhile to further investigate the therapeutic options for serious games in children with a chronic disease, especially when targeting PA behavior. Therefore, the aim of the present review was to study the effectiveness of serious games that promote PA in children with a chronic disease on the outcome of PA, compared to any control group condition. It was hypothesized that children in the serious game groups displayed higher levels of PA than children in the control groups.

## Methods

This review was conducted according to the Preferred Reporting Items for Systematic reviews and Meta-Analyses (PRISMA) guidelines [30] (Multimedia Appendix 1).

### Search Strategy

A comprehensive systematic search was performed using PubMed, EMBASE (Ovid), PsycINFO (Ovid), ERIC (Ovid), CINAHL (EBSCO), and Cochrane Central Register of Controlled Trials (CENTRAL) through the Cochrane Library. The search was conducted in May 2018. Since the use of health games started after 1990, the review was limited to studies published after 1990. We wanted to select chronic diseases with a physical cause. Therefore, a combination of the following constructs was used: Chronic Disease AND Child AND Serious games AND Motor activity AND Intervention study. Only publications in English were included. Multimedia Appendix 2 provides the search string used for each database.

### Screening Process and Eligibility Criteria

The full review screening process was completed by two reviewers (AB and DB), who independently selected the titles and abstracts meeting the inclusion criteria. When an article met the inclusion criteria, full text articles were obtained for closer inspection. To complete the search, the reference lists were checked, and the titles and abstracts of conference proceedings were scanned. When a conference proceeding met the inclusion criteria, the author was contacted and asked for further information on the study process and possible publications. Any unsolved disagreement between the two reviewers was resolved through discussion with a third reviewer (BV).

### Inclusion and Exclusion Criteria

Randomized controlled trials (RCTs) or controlled clinical trials (CCTs) examining the effects of a serious game on PA levels in children with a chronic disease were included. Participants needed to be 6-18 years old. The definition of a chronic disease was based on the criteria by Mokkink et al [3]. We included all serious games that focused on PA behavior and were designed to entertain children with a chronic disease. Serious game interventions fulfilling the criteria of Bergeron [17] were included. These included interventions that (1) had challenging goals, (2) were fun to play and engaging, (3) incorporated some concept of scoring, and (4) provided the player with skills, knowledge, or an attitude that could be applied in the real world. Studies were excluded when PA measurements were not used and when the children, apart from the chronic disease, had a serious intellectual disability. These exclusion criteria were used to increase the comparability of the intervention outcome and population by standardizing the knowledge and understanding to general age-matched education levels.

### Data Extraction

Collected data comprised study, study population, and intervention characteristics. The study characteristics were first author, year of publication, country, study design, type of control group, and follow-up, drop-out, and adherence rates. The primary outcome measure for this review was the level of PA. Objective (eg, accelerometer, pedometer) and subjective (eg, questionnaires, PA diary) PA outcomes were systematically extracted. Other outcomes, including cardiorespiratory endurance, muscular strength, body composition, and quality of life, were also retrieved. Data about the study population (eg, disease, age, gender) and intervention characteristics (eg, type and structure of serious games) were extracted. Missing data were requested from study authors.

### Quality Assessment

Two reviewers (AB and DB) independently assessed the methodological quality and risk of bias criteria (at the study level) of the articles using the Cochrane Collaboration’s tool for assessing the risk of bias [31]. Items were rated as low risk, high risk, or unclear when there were no data to assess the criteria. Items scored as low risk received 1 point. Items scored as high risk or unclear received 0 points. In line with the PRISMA guidelines, an interrater process was adopted, and the degree of agreement was assessed to reduce the risk of bias. Disagreements between the authors on the risk of bias were resolved by discussion, with involvement of a third review author (BV) when necessary.

### Data Synthesis and Statistical Analyses

When possible, data were pooled to assess the combined effects of the studies on PA levels. The mean outcome difference and SD in PA were retrieved from the data of each study. Continuous outcomes are presented as standardized mean difference (SMD) scores with 95% CIs. When indicated, data were converted from measurement in weeks to days, and where moderate and vigorous PA were separate groups, they were combined into a moderate-to-vigorous PA group. Heterogeneity in the effect measures between the studies was assessed using both the Chi-square test and I^2^ statistic [32] and interpreted following the thresholds in the Cochrane Handbook for Systematic Reviews of Interventions, where a *P* value <.1 and/or an I^2^ value >50% may represent substantial heterogeneity [31]. A fixed-effects model was used when heterogeneity was low; otherwise, a random-effects model was applied. The meta-analysis was performed on the latest follow-up point for each study. In addition, a sensitivity analysis based on study quality was conducted. Since Web-based interventions contain game elements other than exergames, we performed a subgroup analysis with respect to the type of intervention. Moreover, we executed a subgroup analysis on diagnosis. The meta-analysis was performed using Review Manager (RevMan) version 5.3 (The Nordic Cochrane Centre, The Cochrane Collaboration, Copenhagen) software. The protocol of this review is available in the International Prospective Register of Systematic Reviews: CRD42018070662.

## Results

### Search Results

**Figure 1:**
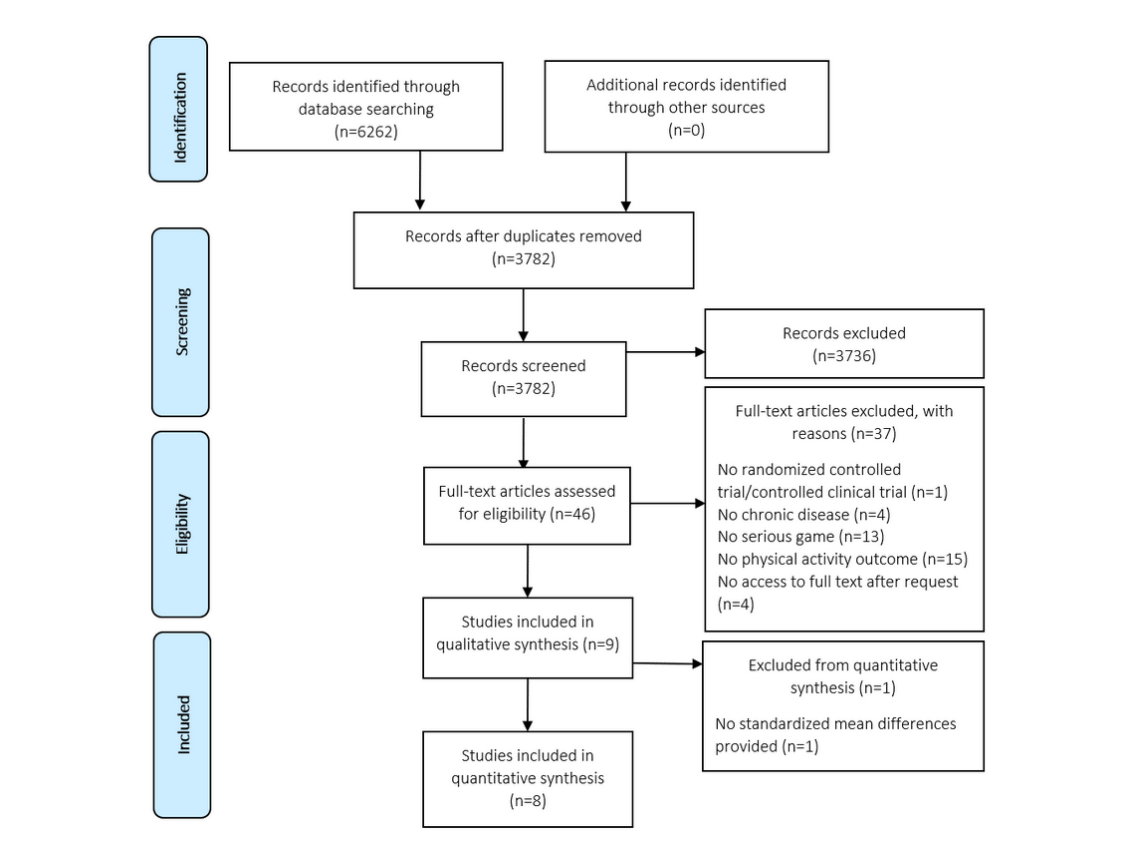
Preferred Reporting Items for Systematic Reviews and Meta-Analyses (PRISMA) flowchart showing the selection procedure for the studies in this systematic review and meta-analysis.

The initial search yielded 6262 publications: 1577 from PubMed, 1939 from EMBASE, 355 from PsycINFO, 362 from ERIC, 1397 from Cochrane Central, and 632 from CINAHL. After duplicates were removed, 3782 articles remained for which the titles and abstract were screened. Finally, 46 full-text articles were assessed for eligibility. Consensus about the inclusion of one study was not reached [18]; after discussion with the third reviewer, this study was included. Eventually, the review included 9 studies, of which 8 could be used in the meta-analysis. The study by Armbrust et al [18] could not be included in the meta-analysis because it used median outcome scores. An overview of the search strategy is provided in Figure 1.

### Methodological Quality Assessment of the Manuscripts

**Figure 2:**
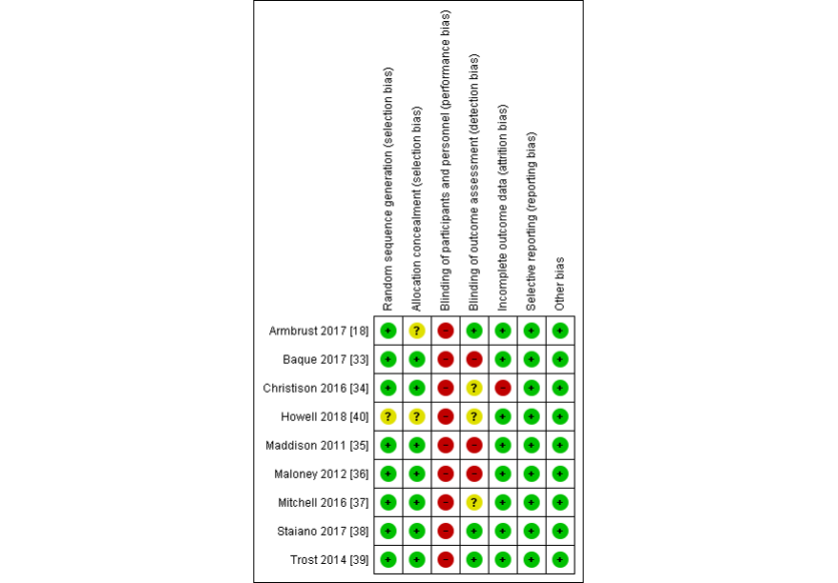
Risk of bias summary of the included studies.

Methodological quality assessment resulted in 98% interrater agreement between the two assessors with a kappa value of 0.98 (95% CI 0.95 to 1.00). The majority of the studies showed no bias in the random sequence generation [18,33-39], allocation concealment [33-39], and complete outcome data [18,33,35-40]. None of the studies fulfilled the criteria of blinding the participants and personnel. In 6 studies, the outcome assessment was not blinded, or the blinding was unclear [33,40]. None of the studies had selective reporting. Of the 9 studies, 8 [18,33,35-40] had complete outcome data and therefore had a low risk of attrition bias. Regarding other forms of bias, commercial sponsorship was investigated. We did not detect any financial conflicts of interest or other forms of bias. The risk of bias is presented in Figure 2.

### Study Characteristics

Table 1 shows the characteristics of the included studies. All 9 studies used a 2-arm RCT design. All studies used an accelerometer to assess PA levels. In addition to the accelerometers, 4 studies measured objective PA using step counts with a pedometer [33,36]. Three studies used a combination of self-reported and objective PA data [18,36,38]. Other collected outcome measures were cardiorespiratory endurance [18,33-37], body composition [34-36,38,39], muscle strength [33,37,40], and health-related quality of life [18,40]. In 6 of 9 studies, the control group received usual care without any additional care [18,33,35-38]. In one study, the control group underwent a family-based intervention [39], and in 2 studies, the control group received education information [34,40]. Studies used different follow-up periods, and the total study duration varied from 12 weeks [37] to 12 months [18].

**Table 1:** Characteristics of the studies included in the systematic review and meta-analysis.

Study	Participants				Intervention		Outcomes		Drop-outs	
Author, year, country, study design	Sample size (n for I^a^, n for C^b^)	Gender (girls), n (%)	Age (years), mean (SD)	Chronic disorder	Treatment	Control	Physical activity measurements	Other outcomes	n for I, n for C	Timing (weeks)
Armbrust, 2017, Netherlands, 2-arm multicenter RCT^c^ [18]	49 (28, 21)	33 (67%)	9.9 (8.7 to 11.3^d^)	Juvenile idiopathic arthritis	Web-based intervention	Supervised group sessions	AM^e^, 7-day activity diary	Cardiorespiratory endurance, quality of life	2, 1	14
Baque, 2017, Australia, 2-arm RCT [33]	60 (30, 30)	26 (45%)	12 (2.5)	Brain injury	Exergame	No intervention (waiting list)	AM, pedometer	Cardiorespiratory endurance, functional muscle strength	4, 3	20
Christison, 2016, USA, 2-arm RCT [34]	84 (60, 24)	46 (58%)	11.1 (1.3)	Obesity	Exergame	Didactic program with family	AM, pedometer	Cardiorespiratory endurance, body composition	24, 8	6^f^
Howell, 2018, USA, 2-arm RCT [40]	94 (63, 31)	43 (55%)	12.7 (1.1)	Cancer survivors	Web-based intervention	Activity monitor and educational materials	AM	Muscle strength, quality of life	10, 6	24
Maddison, 2011, New Zealand, 2-arm RCT [35]	322 (160, 162)	87 (27%)	11.6 (1.1)	Overweight & obesity	Exergame	No intervention (waiting list)	AM	Cardiorespiratory endurance, body composition	20, 12	24
Maloney, 2012, USA, 2-arm RCT [36]	64 (33, 31)	34 (53%)	12.3 (2.4)	Overweight & obesity	Exergame	Pedometers only	AM, pedometer, questionnaire	Body composition, cardiorespiratory endurance	0, 0	12
Mitchell, 2016, Australia, 2-arm RCT [37]	101 (51, 50)	48 (48%)	11.3 (2.5)	Cerebral palsy	Web-based intervention	No intervention (waiting list)	AM, pedometer, questionnaire	Cardiorespiratory endurance, muscle strength	4, 6	20
Staiano, 2017, USA, 2-arm RCT [38]	37 (19, 18)	37 (100%)	15.7 (1.3)	Overweight & obesity	Exergame	No intervention	AM, questionnaire	Body composition	5^g^*	13
Trost, 2014, USA, 2-arm multicenter RCT [39]	75 (34, 41)	41 (55%)	10.0 (1.7)	Overweight &obesity	Exergame	Family-based weight management program	AM	Body composition	3, 3	16

^a^I: intervention group.

^b^C: control group.

^c^RCT: randomized controlled trial.

^d^IQT: interquartile range.

^e^AM: accelerometer.

^f^months.

^g^I + C, not stratified by group.

### Sample Characteristics

A total of 886 participants were included in this review. The sample consisted of 582 children diagnosed with obesity [34-36,38,39], 101 children diagnosed with cerebral palsy [37], 94 survivors of childhood cancer [40], 60 children with a previous brain injury [33], and 49 children diagnosed with juvenile idiopathic arthritis [18]. Per study, the sample size ranged from 37 [38] to 322 patients [35]. Overall, 111 of the 886 participants did not complete the respective study. There were no large gender differences; an almost equal percentage of girls (44.6%; range 27%-100%) and boys (55.4%; range 0%-73%) participated. The age of the participants ranged from 8 to 18 years, with an estimated mean age of 11.8 years (SD 1.7 years).

### Intervention Characteristics

Table 2 presents the PA and technology used during the serious game interventions. The first type of serious game was exergames. Exergames involve PA in gameplay, such as dancing, boxing, and balancing exercises using a Nintendo Wii, PlayStation, or Xbox [33-35,38,39]. The second type of serious game was Web-based educational interventions [18,37,40]. These interventions use puzzles, avatars, and brain twisters to encourage PA.

The duration of the interventions ranged from 10 [34] to 24 weeks [35] with a mean duration of 17.3 weeks (Table 3). In 2 studies, children were instructed to ‘play’ the serious game daily [33,37]; in another 2 studies, children were requested to play the game at least once a week [18,38]. The remaining 5 studies did not specify the frequency of playing [34-36,39,40].

**Table 2:** Description of the physical activity and technology used in the interventions.

Study	Setting	Type of technology	PA^a^ elements	Serious game description
Armbrust et al [18]	Home environment	Web-based application	Arthritis and physical activity education, including barriers, PA benefits, and information about self-efficacy towards becoming more physically active	Films, animations, spoken text, puzzles, brain twisters, and assignments to promote PA; goal setting; email reminders to complete assignments; and a feedback loop to verify whether the child had read the information and finished the assignment. Cognitive behavioral theory was used.
Baque et al [33]	Home environment	Exergame with internet-connected computer and Microsoft Kinect	Gross motor activities combined with cognitive and visual perception and upper limb exercises	Gross motor and daily PA assignments represented on a computer. An example is to use cognitive and visual perception and move the upper limb to solve a mathematical equation. Persuasive elements consisted of feedback and positive reinforcement by parents/guardians.
Christison et al [34]	Research laboratory	Exergame through a PlayStation and Nintendo Wii	Aerobic and muscle strength exercises	A group activity with several games, including aerobic dance, interactive stationary biking, hitting/kicking targets, and boxing. The games used goal setting and documentation of PA in diaries rewarded with small incentives (not specified).
Howell et al [40]	Home environment	Web-based application	Promotion of moderate to vigorous physical activities	The goal was to progress the avatar through various levels on a website. Educational materials, an activity monitor, and access to an interactive website were used to encourage PA via rewards. Points could be redeemed for small prizes (eg, t- shirts, stickers) and/or gift cards.
Maddison et al [35]	Home environment	Exergame through a PlayStation	Promotion of light- to moderate-intensity physical activity	The games were Play3, Kinetic, Sport, and Dance Factory. This was combined with information and education about PA.
Maloney et al [36]	Home environment	Exergame through a PlayStation and Wii	Promotion of physical dancing	Games to encourage dancing.
Mitchell et al [37]	Home environment	Web-based application	Functional gross motor exercises such as sit-to-stand, squatting, and balancing	The Web-based exercises involved upper limb and visual-perceptual games. Examples of active video games are flying a spaceship while squatting and balancing on foam or lunging to shoot a pirate ship with a cannon ball.
Staiano et al [38]	Research laboratory	Exergame through an Xbox 360 console	Encouragement of whole-body movement and moderate-intensity energy expenditure.	Different dance games. Games, songs, dance mode, intensity level, and dance partner were self-selected by the participant.
Trost et al [39]	Schools and young men's Christian associations	Exergame through an Xbox 360 console	Not specified	During the second session, the JOIN for ME program was supplemented with an active sports game. A second active game was provided in week 9 of the JOIN for ME program. No explicit advice or goal was given regarding the use of the active gaming tool.

^a^PA: physical activity.

**Table 3:** Characteristics of the serious game interventions.

Study	Duration (weeks)	Frequency/ intensity	Guidance and supervision	Measurement points	Game adherence
Armbrust et al [18]	14	Weekly/not specified	4 supervised group sessions with parents and children and contact via email with research personnel. Parents were requested to participate.	Baseline, 3 months, and 12 months	Not specified
Baque et al [33]	20	30 minutes per day, 60 hours game play in total	Supervision by a caregiver and assessment of online adherence. Parents were requested to participate.	Baseline and 20 weeks	Mean 17.57 hours (SD 14.9 hours, range 0-46.14 hours) of 'Move it to improve it' (Mitii) training, average of 52.68 logins (SD 39.98 logins)
Christison et al [34]	10	Not specified	10 supervised group sessions, 4 monthly maintenance sessions by a dietitian or counselor, and medical students as facilitators. Parents were requested to participate.	Baseline, 10 months, and 6 months	Not specified
Howell et al [40]	24	Not specified	None	Baseline, 12 weeks, and 24 weeks	Not specified
Maddison et al [35]	24	Not specified	None	Baseline, 12 weeks, and 24 weeks	At 12 weeks, 15.5 minutes a day (SD 26.3 minutes a day); at 24 weeks, 10.2 minutes a day (SD 23.9 minutes a day)
Maloney et al [36]	12	Not specified	2-6 contacts with research personnel over a 20-week period. Contact by e-mail or fax was made if participants did not use the program for 2 weeks.	Baseline and 12 weeks	89 minutes per week over the 12-week period
Mitchell et al [37]	20	30 minutes a day on 6 days a week	Contact with therapists via email, telephone, or video conferencing for encouragement and technical support.	Baseline and 20 weeks	32.4 hours (SD 17.2 hours) of training over the 20-week period, logging in for 24.2 minutes (SD 5.5 minutes) on an average of 77.7 days
Staiano et al [38]	12	60 minutes a day, 3 times a week	Three “Gaming Coaches” were present to supervise the game sessions.	Baseline and 14 weeks	Not specified
Trost et al [39]	16	Not specified	The JOIN for Me program consisted of 16 weekly supervised sessions with groups of children and parents.	Baseline, 8 weeks, and 16 weeks	8 children discontinued the program

In 7 of 9 studies, the intervention was combined with personal guidance from a healthcare provider or researcher [18,33,34,36-39]. In 3 interventions, the guidance consisted of supervised group sessions [18,34,39]. In another 3 studies, one-on-one guidance was provided by a therapist or game coach [33,36,38], and 1 study used digital communication strategies, including email, telephone, or video conferencing [37]. Additionally, only 1 study (Armbrust et al [18]) reported the use of a health behavior theory (cognitive behavioral theory) to support the design of the intervention.

### Main Outcomes

#### Narrative Analysis

Significant between-group differences in PA levels in favor of the intervention group were reported by 2 studies [38,39]. The first study by Trost et al [39] reported statistically significant increases in moderate-to-vigorous (7.4 minutes a day) and vigorous (2.8 minutes a day) PA. In the second study by Staiano et al [38], significant positive effects were observed for self-reported PA levels but not for accelerometer-measured PA. Although most other studies reported significant within-group results in favor of the intervention group [18,33,36,40], these differences were not significant when compared between the study groups.

Body composition was measured in 5 studies [34-36,38,39]. Of these studies, 3 [35,38,39] reported significant end-of-study measurement differences between the intervention and control groups. With respect to cardiorespiratory endurance, there were 6 studies reporting on this outcome [18,33-37]; only the trial by Mitchell et al [37] found significant improvements in the intervention group. Muscle strength was measured in 3 studies [33,37,40], of which 2 studies [33,37] reported a significant increase in muscle strength in favor of the intervention group. Two studies assessed health-related quality of life [18,40] and reported no between-group differences on this outcome measure.

#### Meta-analysis

**Figure 3:**
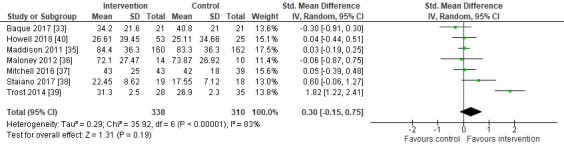
Forest plot for the meta-analysis of moderate to vigorous physical activity (minutes per day).

**Figure 4:**
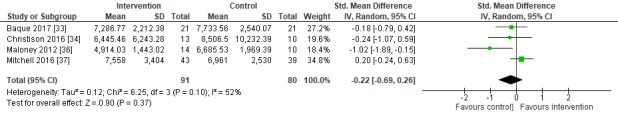
Forest plot for the meta-analysis of physical activity (step counts).

For the primary outcome of PA, accelerometer and pedometer data were extracted to perform a meta-analysis to investigate the effectiveness of serious games on objective PA levels (Figures 3 and 4). The outcome was described as minutes per day of moderate to vigorous PA. The study by Christison et al [34] could not be included in the accelerometer meta-analysis, since this study used only step counts to measure PA. The meta-analysis results showed a positive but nonstatistically significant effect in favor of the intervention group (SMD 0.30, 95% CI –0.15 to 0.75, *P*=.19). Assessment of statistical heterogeneity indicated high study heterogeneity (χ^2^_6_=35.9, *P*<.001, I^2^=83%), which supported the performance of a random effects meta-analysis. In addition to minutes per day of moderate to vigorous PA, we also performed a meta-analysis on step counts. The meta-analysis showed a nonsignificant negative effect on step counts [33,34,36,37], which means that the intervention group had lower step counts than the control group (SMD –0.22, 95% CI –0.69 to 0.26, *P*=.37) with statistical heterogeneity (χ^2^_3_=6.3, *P*=.10, I^2^=52%).

**Figure 5:**
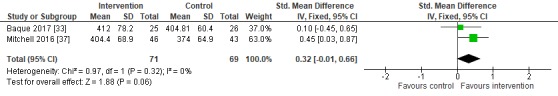
Forest plot for the meta-analysis of cardiorespiratory endurance (6-minute walk test).

**Figure 6:**
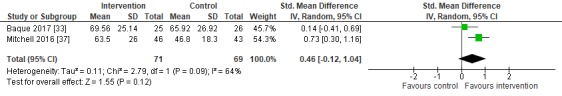
Forest plot for the meta-analysis of functional muscle strength.

**Figure 7:**
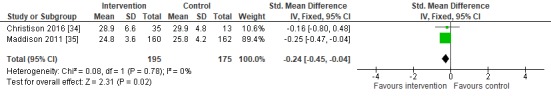
Forest plot for the meta-analysis of BMI.

Regarding other outcomes, 3 other meta-analyses were performed on cardiorespiratory endurance, functional muscle strength, and body composition. The forests plots are provided in Figures 5-7. Compared to the control group, serious games had no significant influence on submaximally tested cardiorespiratory endurance (SDM 0.32, 95% CI –0.01 to 0.66, *P*=.06) or functional muscle strength (SMD 0.46, 95% CI –0.12 to 1.04, *P*=.12). However, there was a significant intervention effect for BMI; the studies reported a reduction in BMI in favor of the intervention group (SMD –0.24, 95% CI –0.45 to –0.04, *P*=.02). A meta-analysis on health-related quality of life could not be performed due to different testing instruments and outcome presentation [18,40].

#### Subgroup and Sensitivity Analysis

Sensitivity analysis was performed for the primary outcome measure PA by excluding the studies with the highest risk of bias. After removal, the direction and magnitude of the SMD did not markedly differ. We also performed two subgroup analyses based on disease stratification (obesity and overweight versus other chronic diseases) and type of intervention (Web-based interventions versus exergame interventions). Additionally, we performed a post-hoc analysis for age by excluding the study by Staiano et al (>15 years) [38]. These analyses showed no significant difference in intervention effectiveness (Multimedia Appendix 3).

## Discussion

This review and meta-analysis showed no significant effects of serious games on PA levels in children with a chronic disease. Only the meta-analysis on BMI showed positive intervention effects, with a significantly greater reduction in BMI in the intervention group. This review is the first to assess the effectiveness of serious games on PA in children with a chronic disease, while former reviews studied the effectiveness of serious games in the promotion of PA levels in healthy children [21-24,26,27,41,42]. Results of these reviews were inconsistent. Some showed significant positive effects on PA [21-23,26,27], another was inconclusive [24], and others did not find positive effects [41,42].

We wondered why the serious games in this review failed to increase PA in children with a chronic disease. A possible explanation can be related to poor adherence rates and low intensity during use. In serious games, it is uncommon to have specific time frames or necessary duration of playtime. Serious games provide (a perception of) autonomy by allowing a child to choose their own exercise levels and own training intensity [43,44]. However, availability and simple promotion of serious games does not automatically lead to improved PA levels [45]. The success of serious games requires explicit instructions and active participation, and children must complete a certain amount of required game content to reach sufficient levels of PA in daily life. In training programs for children, the so-called F.I.T.T. factors (frequency, intensity, time, and type) are recommended [46]. According to these factors, the frequency of training should be at least twice per week for at least 12 weeks, the intensity should be higher than 66% of peak heart rate, and the duration should be between 20 and 60 minutes per session [47]. Of the 9 included studies, 4 studies reported substantial rates of nonuse [34,36-38], which might be the reason for a lack of change in PA behavior. These poor adherence rates are not limited to video games and are recognized as a universal problem in all types of PA exercise interventions [48].

Another factor that may have negatively influenced serious game effects in our review is the absence of information on behavior change techniques in the design of the interventions to enhance PA behavior. In general, behavior change techniques are recommended in the design of complex health service interventions [49] and are important in the promotion of PA [50]. There were only a few studies that mentioned the use of goal setting, rewards, and positive reinforcement to stimulate PA levels [18,33,40]. Other studies paid no or little attention to behavior change techniques [35-39].

Offering a serious game alone is not enough. Serious games should contain support from others in order to increase PA behavior in children. Parents are the most important role model for children and therefore can stimulate their PA participation [51,52]. In the study by Trost et al [39], parents had an active role during the intervention period. This was the study that reported positive effects of the intervention on PA during the assessment of a 16-week family-based pediatric weight management program. Due to the mixed intervention design in this study, it is difficult to tell which part of the intervention was the most important to increase PA. In 4 other studies, the parents had only an administrative role during the intervention period; this role included signing informed consent forms and providing input for data collection such as baseline characteristics and study administration purposes [35,36,38,40].

There are several limitations that need to be addressed. First, in this review we could include only a limited number of studies, almost all of which had small sample sizes. Therefore, it should be noted that the findings with respect to the other outcomes (ie, cardiorespiratory endurance, functional muscle strength, and body composition) must be interpreted with caution since these results were based on only 2 studies. Second, due to limited details about the game structure and exercises included in the serious games, the repeatability of the included studies is low. Third, the interventions were not homogeneous. This might be the consequence of the variation in the primary aim between interventions; some studies focused on a multicomponent intervention to achieve weight loss and increase PA, while others only focused on increasing PA. Last, 5 of the 9 studies [34-36,38,39] addressed overweight and obese participants. It could be that those studies were predominantly focused on body composition, rather than PA, making it difficult to compare those studies.

In line with the findings and limitations of this systematic review, there is a need for more high-quality studies with larger homogeneous sample sizes to better understand the long-term impact of serious games. Furthermore, future research should determine which components, including game duration, intensity, genre, and behavior change techniques, enhance the effectiveness of serious games. Research targeting those components is essential for the development of effective serious games. In addition, studies should aim to determine which strategies are effective to improve game adherence. Studying dose-response relationships between game exposure and PA as the outcome would be useful. Since this review included heterogeneous interventions and samples with a diversity of diseases, future reviews should use a more homogeneous approach.

## Appendix

Multimedia Appendix 1 PRISMA checklist.

Multimedia Appendix 2 Keywords (PubMed version).

Multimedia Appendix 3 Subgroup and sensitivity analyses.

## Abbreviations

AM: accelerometer.
CCT: controlled clinical trial.
PA: physical activity.
PRISMA: Preferred Reporting Items for Systematic Reviews and Meta-Analyses.
RCT: randomized controlled trial.
SMD: standardized mean difference.
