# Supplementary material for: Effectiveness of Serious Games to Increase Physical Activity in Children With a Chronic Disease: Systematic Review With Meta-Analysis
Source: J Med Internet Res. 2020 Apr 1;22(4):e14549. doi: 10.2196/14549 (PMC7160705; doi:10.2196/14549)
Supplement: Multimedia Appendix 2 [file jmir_v22i4e14549_app2.docx]

("Chronic Disease"[Mesh] OR "Diabetes Mellitus"[Mesh] OR "Neoplasms"[Mesh] OR "Leukemia"[Mesh] OR "Overweight"[Mesh] OR "Cystic Fibrosis"[Mesh] OR "Arthritis, Juvenile"[Mesh] OR "Asthma"[Mesh] OR "Attention Deficit Disorder with Hyperactivity"[Mesh] OR "Epilepsy"[Mesh] OR "Kidney Diseases"[Mesh] OR "Cerebral Palsy"[Mesh] OR "Autistic Disorder"[Mesh] OR "Autism Spectrum Disorder"[Mesh] OR "Heart Failure"[Mesh] OR chronic disease*[tiab] OR chronic ill*[tiab] OR chronically ill*[tiab] OR chronic disorder*[tiab] OR chronic health[tiab] OR chronic condition*[tiab] OR diabetes[tiab] OR cancer[tiab] OR overweigh*[tiab] OR obes*[tiab] OR juvenile rheuma*[tiab] OR ADHD[tiab] OR attention deficit disorder*[tiab] OR epilep*[tiab] OR kidney disease*[tiab] OR renal disease*[tiab] OR cystic fibros*[tiab] OR cerebral palsy[tiab] OR asthma*[tiab] OR heart failure[tiab]) AND ("Child"[Mesh:NoExp] OR "Adolescent"[Mesh] OR "Pediatrics"[Mesh] OR "Minors"[Mesh] OR child*[tiab] OR pediatr* OR paediatr* OR peadiatric* OR adoles*[tiab] OR teen*[tiab] OR youth*[tiab] OR schoolchild*[tiab] OR school child*[tiab] OR school*[tiab] OR kid[tiab] OR kids[tiab] OR juvenil*[tiab] OR teen*[tiab] OR pubescen*[tiab] OR puber*[tiab] OR prepubert*[tiab] OR school age*[tiab] OR schoolage*[tiab] OR minor[tiab] OR minors[tiab] OR boy*[tiab] OR girl*[tiab] OR under*age* OR young people[tiab]) AND ("Telemedicine"[Mesh] OR "Telecommunications"[Mesh] OR "Video Games"[Mesh] OR "Internet"[Mesh] OR "Virtual Reality Exposure Therapy"[Mesh] OR "Cell Phones"[Mesh] OR "Computers, Handheld"[Mesh] OR "Mobile Applications"[Mesh] OR "Therapy, Computer-Assisted"[Mesh] OR "Multimedia"[Mesh] OR serious gam*[tiab] OR computer gam*[tiab] OR tele*[tiab] OR internet*[tiab] OR ehealth*[tiab] OR e-health*[tiab] OR m-health*[tiab] OR mhealth*[tiab] OR online[tiab] OR web-based*[tiab] OR mobile health[tiab] OR smartphone*[tiab] OR mobile phone*[tiab] OR mobile*[tiab] OR game*[tiab] OR gaming[tiab] OR exergam*[tiab] OR gamification*[tiab] OR app[tiab] OR apps[tiab] OR applicat*[tiab] OR persuasive technolog*[tiab] OR video*[tiab] OR electronic*[tiab] OR virtual*[tiab] OR digital*[tiab] OR multimedia[tiab]) AND ("Motor Activity"[Mesh] OR "Locomotion"[Mesh:NoExp] OR "Exercise"[Mesh] OR "Physical Fitness"[Mesh] OR "Physical Examination"[Mesh] OR "Physical Therapy Modalities"[Mesh] OR "Exercise Therapy"[Mesh] OR "Exercise Movement Techniques"[Mesh] OR "Sports"[Mesh] OR sport*[tiab] OR walk*[tiab] OR swim*[tiab] OR running*[tiab] OR jogging[tiab] OR motor[tiab] OR exercis*[tiab] OR physical activ*[tiab] OR body movement*[tiab] OR movement therap*[tiab]) AND ("Clinical Trial" [Publication Type] OR "Controlled Clinical Trial" [Publication Type] OR "Randomized Controlled Trial" [Publication Type] OR "Controlled Clinical Trials as Topic"[Mesh] OR "Random Allocation"[Mesh] OR "Double-Blind Method"[Mesh] OR "Single-Blind Method"[Mesh] OR random*[tiab] OR clinical trial*[tiab] OR RCT[tiab] OR trial[tiab]) NOT ("Case Reports" [Publication Type] OR "Letter" [Publication Type] OR "Editorial" [Publication Type] OR case report*[tiab] OR case stud*[tiab]) AND (english[Language] OR dutch[Language] OR german[Language])
