# Supplementary material for: Effectiveness of Serious Games to Increase Physical Activity in Children With a Chronic Disease: Systematic Review With Meta-Analysis
Source: J Med Internet Res. 2020 Apr 1;22(4):e14549. doi: 10.2196/14549 (PMC7160705; doi:10.2196/14549)

Multimedia Appendix 2: Subgroup and sensitivity analyses

Sensitivity analysis physical activity, minutes per day
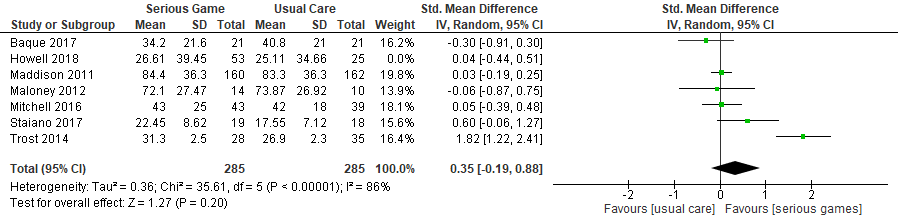


Subgroup analysis physical activity (minutes per day) stratified by intervention (exergame/web-based intervention) and diagnosis (obesity/other diseases)


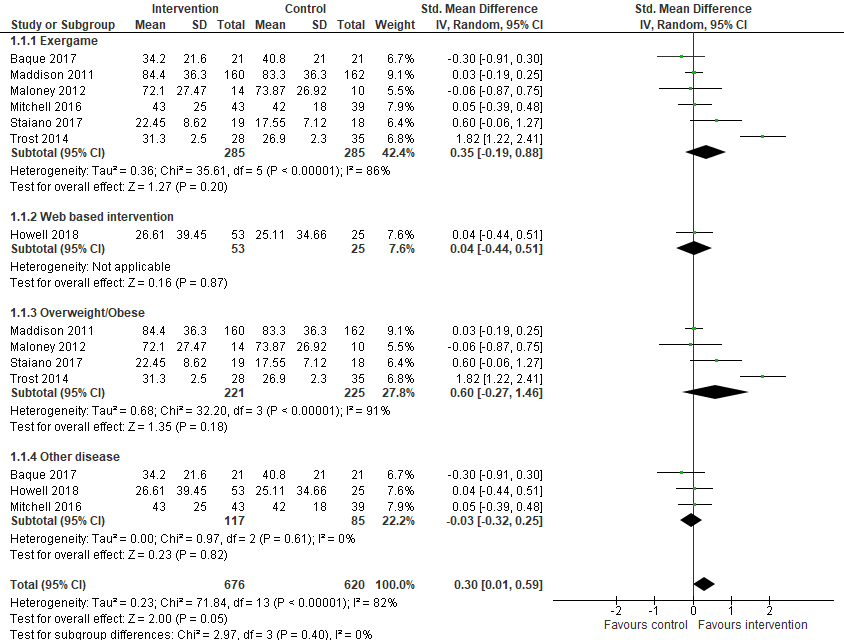


Sensitivity analysis physical activity, step counts
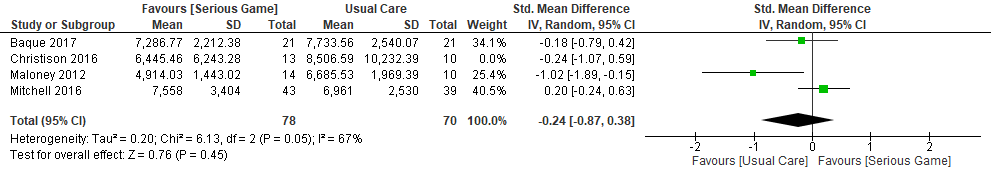


Subgroup analysis physical activity (step counts) stratified by diagnosis (obesity/other diseases)
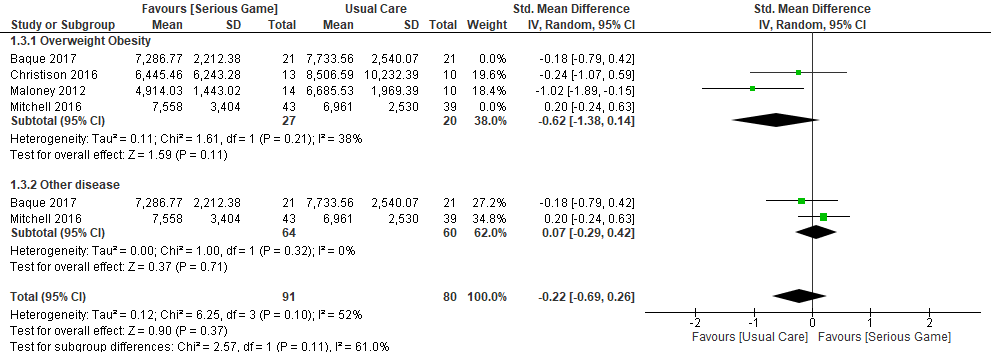


Post-hoc analysis for age on physical activity (minutes per day)


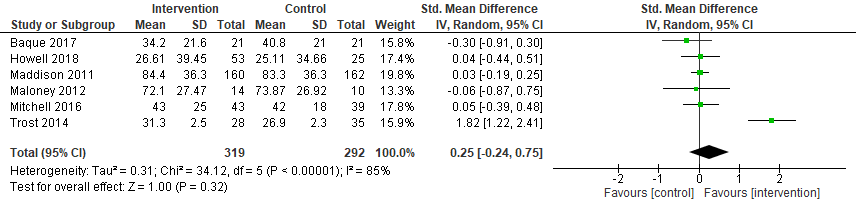

Supplement: Multimedia Appendix 3 [file jmir_v22i4e14549_app3.docx]
